# Physiology-Guided Resuscitation: Monitoring and Augmenting Perfusion during Cardiopulmonary Arrest

**DOI:** 10.3390/jcm13123527

**Published:** 2024-06-16

**Authors:** Samuel Bernard, Raymond A. Pashun, Bhavya Varma, Eugene Yuriditsky

**Affiliations:** Division of Cardiology, New York University Grossman School of Medicine, New York, NY 10016, USA; samuel.bernard@nyulangone.org (S.B.); raymond.pashun@nyulangone.org (R.A.P.);

**Keywords:** cardiopulmonary arrest, cardiopulmonary resuscitation, hemodynamics, resuscitative transesophageal echocardiography, extracorporeal cardiopulmonary resuscitation, mechanical CPR

## Abstract

Given the high morbidity and mortality associated with cardiopulmonary arrest, there have been multiple trials aimed at better monitoring and augmenting coronary, cerebral, and systemic perfusion. This article aims to elucidate these interventions, first by detailing the physiology of cardiopulmonary resuscitation and the available tools for managing cardiopulmonary arrest, followed by an in-depth examination of the newest advances in the monitoring and delivery of advanced cardiac life support.

## 1. Introduction

In the mid-18th century, there was a growing interest in the development of cardiopulmonary resuscitation (CPR), which is when drowning became a great public health issue. Initially, the focus was primarily on ventilation and airway management, with some proposed methods including rolling the victim over a barrel or hoisting them onto a trotting horse [1]. In the late 19th century, open cardiac massage drew attention to cardiac compression as a means to restore circulation. This was initially attempted on animals and subsequently expanded to humans; however, it was restricted to experienced personnel in the operating room or hospital setting. Closed cardiac massage followed soon thereafter and was successfully performed on eight humans. This set the stage for the initial development of closed-chest CPR in the mid 20th century, with the restoration of spontaneous circulation (ROSC) in 14 of 20 cardiac arrest patients utilizing chest compressions, external defibrillation, and mouth-to-mouth ventilation [2].

**Figure 1:**
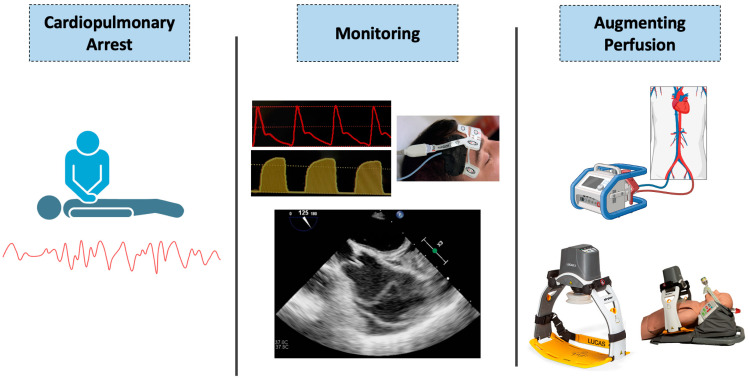
Illustration of some of the available tools used for monitoring and augmenting perfusion in cardiopulmonary arrest. Created with BioRender.com, accessed on 23 April 2024. LUCAS 3 device reproduced with permission from Stryker (Portage, MI, USA). EleGARD device as part of the Automated Head/Thorax Up Position CPR system reproduced with permission from AdvancedCPR Solutions (Edina, MN, USA).

Despite representing the foundation of present-day CPR, chest compressions are inherently inefficient, providing less than 25% of normal cardiac output under the best circumstances [3]. As adequate myocardial oxygen delivery is a fundamental determinant of survival following cardiac arrest, several related physiologic targets have been established to optimize CPR quality [4,5,6]. Recent decades have seen important developments in CPR monitoring technologies aimed at enhancing resuscitation. Beyond end-tidal carbon dioxide (ETCO_2_) and invasive blood pressure monitoring, cerebral oximetry and transesophageal echocardiography (TEE) have been added to the armamentarium with emerging data [7,8]. A better understanding of CPR physiology has led to the development of several devices with the aim of modulating intrathoracic pressure, increasing cardiac output, and improving intra-arrest hemodynamics [9,10]. Extracorporeal cardiopulmonary resuscitation (eCPR) has gained considerable attention over the last several years with a potential role in select circumstances [11]. In this manuscript, we review the existing technologies and developments designed to monitor and augment hemodynamics during cardiac arrest resuscitation (Figure 1). 

## 2. The Physiology of Cardiopulmonary Resuscitation

Modern closed-chest CPR was described by Kouwenhoven and colleagues in 1960 [2]. The authors postulated that the forward flow of blood was generated via direct cardiac compression between the sternum and vertebral column. However, subsequent experimental evidence suggested the heart may function as a passive conduit—blood flows as the intrathoracic pressure exceeds that of the extrathoracic vasculature [12]. To date, the specific mechanism driving blood flow during CPR is inconclusive. Indeed, the prevailing mechanism may differ between patients based on anatomical variations and compression momentum [13]. Here, we review several mechanisms governing blood flow during resuscitation, the existing evidence base, and additional physiologic implications. 

### 2.1. The Cardiac Pump Model

Comparable to an internal cardiac massage, chest compressions were hypothesized to squeeze the ventricles, thereby ejecting blood into the great arteries [2]. Myocardial relaxation during the decompression phase would allow for ventricular filling analogous to diastole. This intuitive description followed that of the cardiac cycle and was accepted despite a lack of supporting evidence [14]. Decades after Kouwenhoven’s initial hypothesis, several animal and human studies provided support for the theory. 

Feneley et al. induced cardiac arrest in dogs and performed two-dimensional echocardiograms during manual CPR. With high-impulse CPR, their group observed the opening of the aortic valve during the compression phase and closure during release. Left ventricular deformation was observed during sternal compression with concomitant mitral valve closure [13]. Performing TEE on 18 patients suffering cardiac arrest, Redberg and colleagues witnessed a reduction in ventricular cavity size with compressions and mitral valve opening during release [15]. The observation of a decrease in ventricular size associated with aortic valve opening and mitral valve closure implied stroke volume was derived from ventricular deformation during the compression phase of CPR. Accordingly, the heart functioned as more than a “passive conduit”, as was predicted with the thoracic pump model [12,13,15].

### 2.2. The Thoracic Pump Model

In 1976, Criley et al. reported on several patients suffering ventricular fibrillation (VF) during coronary angiography. Three patients remained conscious for up to 39 s by forceful coughing, which maintained an aortic systolic pressure in excess of that subsequently delivered by CPR [16]. As the heart was not being compressed, it was thought to function merely as a conduit; variations in intrathoracic and abdominal pressure were thought to propel blood from the intrathoracic vasculature into the systemic circulation. 

To better define this model, catheters were inserted into the ventricles, intrathoracic, and extrathoracic vasculature of 15 dogs with the subsequent induction of cardiac arrest. During CPR, pressures within the left ventricle (LV), aorta, right atrium, and pulmonary artery were identical and equal to the intrathoracic pressure estimated by an esophageal balloon [14]. Maneuvers elevating the intrathoracic pressure (i.e., abdominal binding) improved the aortic systolic pressure as well as carotid flow. The drop in intrathoracic pressure between compressions would be responsible for venous flow into the right ventricle [12]. Inconsistent with this model is the aforementioned observation of mitral valve closure during thoracic compression [15]. However, this phenomenon may not be universal among all patients undergoing CPR [17]. 

Ultimately, it is accepted that both models may be involved in the generation of blood flow. The chest wall anatomy, location of chest compressions, and whether or not a mechanical compression device is used may determine the dominant mechanism. Several additional theories, in part variations on the discussed themes, have since been postulated; however, their clinical relevance is less clearly defined [10].

### 2.3. Microcirculation in Cardiac Arrest 

Microcirculation, or the terminal vascular network of the systemic circulation, is responsible for the regulation of blood flow, tissue perfusion, and oxygen delivery [18]. Its direct assessment involves the use of hand-held sublingual microscopy, allowing for the quantitative and semi-quantitative description of parameters, including the microcirculatory flow index and proportion of perfused vessels. However, simpler surrogates, including the capillary refill time (CRT), the central venous-to-arterial carbon dioxide difference, and skin mottling, are used in clinical practice to assess microvascular blood flow [18]. In shock states, the two components of the circulation may be discordant such that the restoration of macrocirculatory flow as assessed by the arterial pressure or cardiac index may not translate into improved tissue perfusion [19].

While studies have described macrocirculatory indices such as coronary perfusion pressure (CPP) and diastolic arterial pressure, less is known about the role of microcirculation in cardiac arrest [20]. In a porcine model of cardiac arrest, global hemodynamic variables poorly correlated with microcirculatory flow assessed via sublingual microscopy [21]. However, this finding was not consistent across studies [22]. In an experimental model of ventricular fibrillation, the administration of epinephrine, an intervention known to increase CPP, was shown to significantly reduce microcirculatory flow [20]. Further, microcirculatory flow has been shown to predict outcomes in an experimental model of cardiac arrest with good sensitivity and specificity [22].

Following ROSC, microcirculatory alterations improve but may not normalize. A systemic inflammatory response mediated by ischemia and reperfusion injury is thought to contribute to these abnormalities [23]. Higher sublingual microcirculatory flow post-arrest may be associated with survival, while persistent alterations are associated with organ failure and death independent of systemic hemodynamics [24,25]. Presently, the literature surrounding microcirculation in cardiac arrest is limited to experimental models and small observational studies. While the results are both promising and interesting, much remains to be learned. Whether interventions aimed to restore microcirculation during and after cardiac arrest improve outcomes requires further exploration [26]. 

### 2.4. Additional Physiologic Considerations 

Augmenting CPP (the pressure difference between the aorta and right atrium during relaxation) and cerebral blood flow are the primary objectives of CPR [6]. Subsequently discussed approaches to CPR and mechanical resuscitative devices rely on the aforementioned models of flow to enhance systemic perfusion. However, cerebral perfusion may be negatively impacted by standard CPR on the basis of high-pressure venous waves and depressed venous return during active chest compressions [27]. Whether alternative approaches to cerebral monitoring and resuscitation can improve outcomes in cardiac arrest remains an important question. This forms the basis of the development of newer perfusion methods used to enhance cerebral blood flow (e.g., impedance threshold, active compression-decompression, and automated head/thorax-up positioning devices) and deliver “neuroprotective CPR”. 

In addition to its role in gas exchange, positive pressure ventilation has a distinct impact on circulatory function. Excessive intrathoracic pressure may limit venous return and generate impedance to right ventricular ejection. Accordingly, proper ventilation and the modulation of intrathoracic pressures may impact hemodynamics during resuscitation [9]. 

Finally, multiple serum and cerebrospinal fluid (CSF) biomarkers have been investigated during and immediately after cardiac arrest in order to better prognosticate mortality and neurologic recovery. Increasingly, these values have been utilized in a decision-making capacity, including that of coronary angiography and eCPR. While an exhaustive discussion of prognostication is outside the scope of this review, lactate and phosphate levels are worth briefly touching upon.

Serum lactate levels are widely utilized as markers of systemic hypoperfusion and tissue hypoxia, with greater values indicative of higher degrees of cellular injury. Absolute lactic acid values and lactate clearance have both been evaluated in critically ill patients and demonstrated associations with mortality [28,29,30]. However, in those with cardiac arrest, the relationship of early lactate levels to patient outcomes has been more inconsistent and inconclusive [31,32,33,34,35]. There has been an even greater paucity of data to guide prognosis after eCPR, with different proposed cutoff values and clearance rates; however, in general, higher lactate levels have been correlated with unfavorable outcomes and mortality in this cohort [33,36,37].

Phosphorus levels have also been examined as a prognostic marker in patients with cardiopulmonary arrest. Phosphorus has been recognized as an integral component in multiple cellular processes, including signal transduction and energy exchange. Multiple studies have demonstrated associations between higher initial post-ROSC phosphate levels with worse neurologic outcomes and greater mortality [38,39]. Hypotheses for this elevation include transcellular shifts and cellular damage. Interestingly, it has also been implicated as an important contributor to acidosis following cardiac arrest [40].

## 3. Monitoring Perfusion during Cardiopulmonary Resuscitation—Current Strategies

Coronary perfusion pressure (CPP), end-tidal carbon dioxide (ETCO_2_), and diastolic blood pressure (DBP) are commonly monitored during CPR. The literature has shown that these parameters can be leveraged to augment resuscitation efforts in a physiology-guided fashion. Updated guidelines from the American Heart Association mention these parameters in varying capacities for modern resuscitation efforts [41]. The practical strengths and limitations of each parameter are described below. 

### 3.1. Coronary Perfusion Pressure (CPP)

The aim of CPR is to maintain coronary and cerebral perfusion for the purpose of ROSC and to promote neurologically favorable outcomes. Myocardial perfusion can be evaluated using the CPP, calculated as the gradient between the aorta and right atrium during “CPR diastole” [6,42]. Small animal and human studies began investigating the value of CPP in the 1980s, noting a direct correlation with myocardial blood flow [43,44,45,46]. In 1990, Paradis et al. performed CPP monitoring in 100 human subjects during CPR through the use of pressure catheters inserted in the right atrium and aortic arch [6,47,48,49]. It was noted that increasing CPP values correlated with increased success of ROSC, thereby providing real-time feedback on the quality of CPR. The initial mean CPP was 1.4 mmHg for those who did not achieve ROSC versus 18.4 mmHg for those who did [6]. Moreover, a maximal initial CPP of 15 mmHg or greater had a positive predictive value of 0.57 (i.e., continued resuscitation efforts would achieve ROSC more than half the time) [6]. Conversely, patients with an initial CPP of 0 mmHg or less never achieved ROSC [6]. 

The aforementioned demonstrates the utility of CPP in CPR in that it provides real-time feedback to the resuscitation team regarding myocardial blood flow and prediction of resuscitation success [42]. However, its invasive nature makes it impractical for most scenarios [42]. Current practices do not typically target this metric but instead target indices and variables that have been correlated with CPP, such as ETCO_2_ and diastolic blood pressure. 

### 3.2. End Tidal Carbon Dioxide (ETCO_2_)

Carbon dioxide (CO_2_) is produced primarily during aerobic metabolism, returned to the lungs via the venous system, and eliminated through alveolar ventilation [50,51]. During exhalation, CO_2_ can be measured with capnography. ETCO_2_ refers to the concentration of exhaled CO_2_ at the end of phase III of the capnogram (representing the emptying of alveoli) [51]. Cardiopulmonary arrest (CPA) causes an abrupt decrease in circulation and CO_2_ transport, and thereby, CO_2_ expulsion, resulting in a dramatic drop in ETCO_2_ [50]. This was first described by Kalenda, who identified that ETCO_2_ decreased with compressor fatigue and increased substantially upon achieving ROSC [52]. Numerous studies thereafter have demonstrated the utility of ETCO_2_ during CPR, including positive associations with cardiac output, myocardial blood flow, CPP, and cerebral perfusion pressure [51,53,54,55]. ETCO_2_ has been described as the best measure of ROSC during CPR, given it can precede a palpable pulse [50,51,56]. It also helps monitor the effectiveness of pulmonary perfusion and, therefore, chest compressions [50,51,57,58]. In fact, a multicenter study of more than 500 patients showed a statistical association between ETCO_2_ and chest compression depth [57]. Moreover, numerous studies show ETCO_2_ > 10 mmHg as a “cut off” value for increased likelihood of achieving ROSC [51,59,60,61,62]. ETCO_2_ can also predict the futility of ongoing resuscitation efforts. In a prospective study of 737 out-of-hospital arrests, the inability to increase ETCO_2_ over 14.3 mmHg after 20 min of CPR had 100% sensitivity and specificity for failure to achieve ROSC [63]. 

The American Heart Association 2020 CPR guidelines state that “targeting compressions to an ETCO_2_ value of at least 10 mm Hg, and ideally 20 mm Hg or greater, may be useful as a marker of CPR quality. An ideal target has not been identified” [64]. Indeed, targeting a specific ETCO_2_ value for ROSC remains an ongoing knowledge gap in light of the fact that the majority of studies to date remain observational [64]. Despite this, ETCO_2_ is a powerful, non-invasive, easily accessible tool with reproducible data that can be used to assist in physiology-guided resuscitation [51,65]. 

Notably, ETCO_2_ has practical limitations in its use. It is most effectively utilized with an advanced airway, as the lack of a good seal on the bag-valve mask or secretions in the sampling tube can make colorimetric ETCO_2_ unreliable [51]. ETCO_2_ values may also be altered in patients with pre-existing lung disease and have been demonstrated to be affected by the administration of IV epinephrine and sodium bicarbonate [51]. While acknowledging these relatively minor limitations, ETCO_2_ is an invaluable tool for the resuscitation team to have rapidly available, real-time feedback during CPR. Unfortunately, even with incorporation into guidelines, in contemporary practice, this tool is not being used to its full extent. In a 2016 review of 21,375 index events from the American Heart Association Get With the Guidelines Resuscitation Registry, only 4% of patients with an invasive airway present at the time of CPA underwent ETCO_2_ monitoring [66]. Similarly, in one study of pediatric patients at two large urban academic emergency rooms, ETCO_2_ was only used in 13% of children [67]. 

### 3.3. Diastolic Blood Pressure (DBP)

The American Heart Association 2020 CPR guidelines also include diastolic blood pressure monitoring during CPR as an updated recommendation in pediatric populations [64]. They state it is reasonable to use diastolic blood pressure to assess CPR quality, and “although ideal blood pressure targets during CPR are not known, diastolic blood pressure is the main driver of coronary blood flow and may be used to guide interventions…” [64]. This is based primarily on a study of 164 children demonstrating that a maintenance of a mean DBP ≥ 25 mmHg and ≥30 mmHg during CPR for infants and children ≥ 1 year old, respectively, was associated with survival with favorable neurological outcomes [68].

Despite its theoretical benefits for monitoring, the use of DBP for monitoring perfusion during CPA for adults is limited. One study of 104 patients from Finland demonstrated that chest compression rates of 100–120 per minute combined with a chest compression depth > 60 mm were associated with DBP > 30 mmHg for both femoral and radial measurements [69]. Notably, while an often-cited limitation of the use of DBP monitoring is the necessity of an arterial line, prior studies have suggested that approximately 59% of in-hospital cardiac arrest (IHCA) occurs in patients in the intensive care unit [70]. 

### 3.4. Utilizing Indicators of Perfusion in Practice

Overall, the American Heart Association 2020 CPR guidelines remain vague regarding specific targets for monitoring parameters, which is reasonable given the lack of robust, randomized human data during CPR. Based on expert consensus, prior guidelines recommend maintaining CPP > 20 mmHg, DBP > 25 mmHg, and ETCO_2_ > 20 mm Hg [3]. It is reasonable to focus efforts primarily on using ETCO_2_ monitoring and feedback during CPR during the time of CPA given its ease of initiation, the fact that invasive catheter placement is not required for its use, and that it has the most supportive human data. Notably, there are limited data comparing the efficacy of all three metrics; however, a recent animal study of 60 subjects suggests DBP predicts survivorship better than ETCO_2_ when conducting CPR [71].

## 4. Monitoring Perfusion during Cardiopulmonary Resuscitation—Novel Strategies

While invasive hemodynamics theoretically provide the greatest amount of information regarding cerebral, coronary, and systemic perfusion, they are also impractical in most cardiac arrest scenarios. Other less invasive alternatives have been suggested to monitor perfusion during CPR, including regional cerebral oximetry and transesophageal echocardiography.

### 4.1. Regional Cerebral Oximetry

Near-infrared spectroscopy (NIRS) allows for portable, continuous, and non-invasive monitoring of the regional hemoglobin oxygen saturation (rSO_2_) in the brain [72,73]. An adhesive probe equipped with a light emitter and detector is applied to the scalp near the frontal cortex. Light in the 650–950 nm range penetrates the skull and provides a percentage estimate of oxygenated hemoglobin in the superficial cortical region. As approximately 70% of the blood volume is in the venous compartment, normal rSO_2_ values range from 60–80% [73]. Lower values may be encountered in cases of reduced cerebral blood flow or oxygen content (i.e., hemoglobin concentration or saturation) and reflect the cerebral oxygen supply–demand balance [72]. This approach to regional circulatory monitoring has been applied to cardiac arrest and may allow for the assessment of CPR quality, the prediction of ROSC, and survival with favorable neurological outcomes [7,73].

Multiple studies have demonstrated an association between higher rSO_2_ values and ROSC, as well as favorable clinical outcomes [74,75]. However, there is significant overlap in values. In one multicenter study of 504 patients with IHCA, those surviving with good outcomes had a higher mean rSO_2_ than those with poor neurological outcomes (56% ± 10% vs. 44% ± 13%) [7]. At extremes, specificity is high; a 25% cut-off is highly predictive of no ROSC, while rSO_2_ > 65% is highly predictive of ROSC [73].

While compelling, the evidence base supporting NIRS as a tool to monitor CPR quality is limited [74,75]. In a study of 34 patients, the use of a mechanical CPR device, demonstrated to improve compression quality, was associated with higher rSO_2_ when compared to manual CPR [75]. Several reports have established a decrease in rSO_2_ with the loss of pulses, a rise in rSO_2_ with initiation of chest compressions, and even further increases with ROSC [76]. These associations suggest that rSO_2_ may reflect hemodynamic changes and cerebral blood flow. 

Cerebral oximetry monitoring and ETCO_2_ have similar diagnostic characteristics in predicting ROSC [77]. However, capnography is influenced by ventilatory parameters, the presence (or absence) of an advanced airway, underlying lung disease, and drug administration [73]. Accordingly, future studies may identify a specific role for cerebral oximetry in perfusion monitoring during CPR.

### 4.2. Transesophageal Echocardiography (TEE)

Transthoracic echocardiography (TTE) has played an increasingly important role in the diagnosis, management, and prognostication of patients who experience CPA. However, TTE suffers from a variety of technical limitations during advanced cardiac life support (ACLS), including limited acoustic windows and motion artifacts during ongoing chest compressions. Observational studies have also suggested that the use of surface cardiac ultrasound may result in longer compression pauses (compared to standard ACLS) without clear benefits in mortality, despite the ability to identify immediately reversible causes of cardiac arrest [78,79,80].

**Figure 2:**
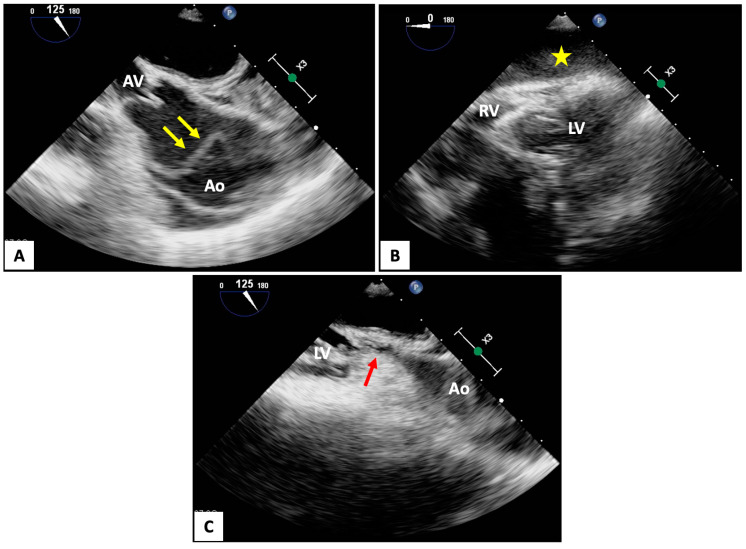
(**A**,**B**) Focused transesophageal echocardiography (TEE) images demonstrating a patient with a type A aortic dissection (yellow arrows) complicated by hemopericardium with tamponade (yellow star). (**C**) The area of maximal compression can be seen at the aortic root (red arrow). Ao, aorta; AV, aortic valve; LV, left ventricle; RV, right ventricle.

Focused TEE has been proposed as an alternative to TTE to obviate some of the aforementioned issues. Due to its retrocardiac position, TEE can provide continuous monitoring and excellent cardiac visualization throughout the entirety of a cardiac arrest without the assortment of technical limitations suffered by TTE. Even in ideal imaging conditions, TEE is known to have greater diagnostic accuracy than TTE for many of the reversible etiologies of a cardiac arrest, including tamponade (particularly if localized), pulmonary embolism, aortic dissection, and mechanical complications due to acute myocardial infarction (Figure 2A,B). TEE can also facilitate the transition to (and cannulation of) eCPR. Importantly, TEE does not seem to alter the natural cadence of CPR. In one retrospective analysis evaluating 25 cardiac arrests (constituting 139 pulse checks), TEE demonstrated shorter durations of pauses during pulse checks (9 s; 95% confidence interval [8–14 s]) as compared to TTE (19 s; 95% confidence interval [16–22 s]) and no significant difference compared to manual pulse checks (11 s; 95% confidence interval [8–14 s]) [81]. At present, the major limitations of TEE are that it requires an established airway (i.e., endotracheal intubation), there are limited numbers of practitioners with TEE experience who are readily available, and positioning of the imager and echocardiography machine may be challenging and has yet to be integrated into previously established CPR team protocols. However, studies have demonstrated an average time of 7–12 min for obtaining the first TEE image during the intra-arrest portion of cardiac arrest [8,15,82]. Further, with dedicated pre-training interventions (particularly in those with prior TTE experience), there are accelerated learning curves in physician familiarity and the use of TEE [83]. 

TEE has also been proposed as a novel method for improving ACLS and overall systemic perfusion through the identification of the area of maximal compression (AMC) during CPR (Figure 2C). Current American Heart Association guidelines recommend chest compressions take place in the center of the lower half of the victim’s sternum [41]. In theory, this is supposed to result in direct ventricular compression, thereby simulating cardiac ejection and generating cardiac output. However, two radiographic studies have suggested that in 46–80% of patients, the structures located in this location are the left ventricular outflow tract (LVOT), aortic valve, aortic root, or ascending aorta [84,85]. TEE appears to corroborate these findings. In a prospective observational study of 34 patients with nontraumatic cardiac arrest, the AMC was located at the aorta in 59% of patients and LVOT in 41% of patients [86]. Similarly, in a prospective study of 33 patients with out-of-hospital cardiac arrest (OHCA) who underwent TEE imaging during CPR, the AMC was identified over the aortic root or LVOT in 53% of cases [8]. As such, current hand placement for chest compressions may paradoxically impede cardiac output.

Few studies have examined the hemodynamic or mortality implications of the AMC location. Swine models have been promising. One such study examined the effect of AMC location on 26 pigs with ventricular fibrillation who were randomized to chest compressions at a “traditional” location (at the aortic root) versus over the center of the LV. Five pigs who received LV-centered compressions achieved ROSC compared to 0 when directed over the aortic root [87]. A separate study measured invasive hemodynamic parameters in 32 swine randomized to standard chest compression versus LV chest compressions. The latter group achieved significantly greater ETCO_2_, mean arterial blood pressure, and cerebral blood velocity. While not statistically significant, 17% of pigs achieved ROSC in the LV chest compression group as compared to 0% in the standard chest compression cohort [88]. In a single swine model utilizing real-time TEE to direct the location of chest compressions, Teran et al. demonstrated that when compressions were directed over the LV (as opposed to the LVOT), aortic pressures and ETCO_2_ were higher [89]. Moreover, the LVOT remained patent with intermittent aortic valve opening during chest compressions. In humans, data are sparse. In one retrospective study of 19 patients who received TEE during CPR, it was noted that LVOT opening was the only variable associated with ROSC [82].

## 5. Augmentation of Circulatory Support

**Figure 3:**
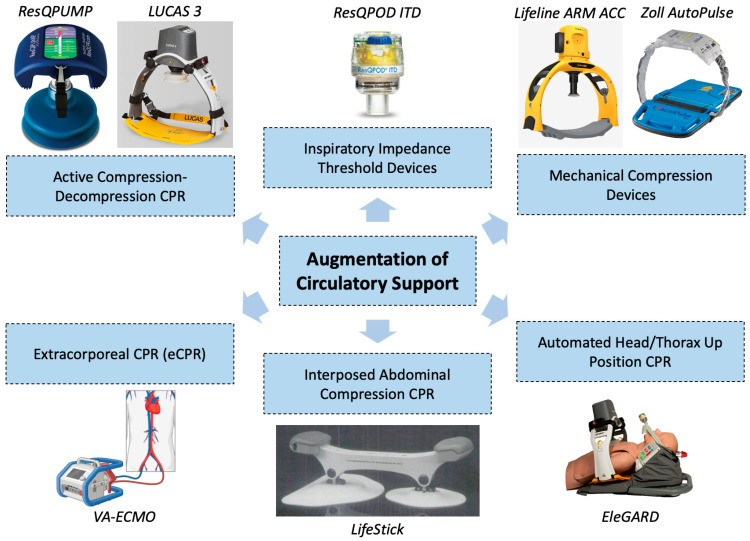
Different methods that have been evaluated for the augmentation of standard cardiopulmonary resuscitation. *CPR*, cardiopulmonary resuscitation. ResQPUMP reproduced with permission from Zoll Medical Corporation (Chelmsford, MA, USA). ResQPod ITD reproduced with permission from Zoll Medical Corporation (Chelmsford, MA, USA). Autopulse device reproduced with permission from Zoll Medical Corporation (Chelmsford, MA, USA). LUCAS 3 device reproduced with permission from Stryker (Portage, MI, USA). Lifeline ARM ACC reproduced with permission from Defibtech, LLC (Guilford, CT, USA). EleGARD device as part of the Automated Head/Thorax Up Position CPR system reproduced with permission from AdvancedCPR Solutions (Edina, MN, USA). Lifestick reproduced with permission from Lurie and Lindner [90].

The optimization of coronary and cerebral perfusion during cardiac arrest to promote ROSC and minimize hypoxic–ischemic brain injury is primarily achieved through high-quality chest compressions. Multiple approaches and adjunctive therapies to augment perfusion have been investigated in an effort to increase the likelihood of achieving ROSC and improve outcomes (Figure 3, Table 1). These therapies include various mechanical devices, respiratory valves, adjustments to patient positioning, and (most recently) venoarterial extracorporeal membrane oxygenation (VA-ECMO).

### 5.1. Standard Methods

Early CPA research was primarily focused on optimizing the various aspects of “standard CPR” as we know it today. Initial studies demonstrated poor outcomes associated with suboptimal perfusion due to inadequate performance of resuscitative measures. This included hyperventilation during CPR (associated with increased intrathoracic pressure, resulting in decreased preload and CPP) and poor-quality chest compressions (associated with inadequate CPP and low rates of ROSC) [6,91]. Ensuring appropriate ventilation and optimizing the rate and depth of chest compressions while minimizing interruptions are essential to improving outcomes [92,93]. 

### 5.2. Active Compression–Decompression CPR

Active compression–decompression CPR (ACD-CPR) is an alternative approach to chest compressions that utilizes a suction device with the intention of converting the passive chest wall expansion during traditional CPR to active expansion, enhancing venous return. The device design was based on out-of-hospital cardiac arrest (OHCA) data, where increased chest wall recoil velocity during traditional CPR was associated with improved survival and favorable neurologic outcomes. In animal and human studies, ACD-CPR is associated with improved cerebral perfusion, myocardial perfusion, and cardiac output [94,95,96,97,98]. Despite this favorable hemodynamic effect, in a predominantly OHCA patient population, there has been no consistent benefit of ACD-CPR on mortality, survival to discharge, survival at 1 year, or neurologic outcome [99,100,101,102]. Additionally, there was no observed increased risk for major complications from the device such as rib fractures, hemothorax, or pneumothorax. One multicenter randomized trial of OHCA patients demonstrated improved survival with ACD-CPR; however, this was confounded by the utilization of an impedance threshold device (described below) in the treatment arm [103]. Further clinical trials of ACD-CPR with an impedance threshold device in OHCA have demonstrated improved survival at a 1-year follow-up with favorable neurologic status [104,105]. This is suggestive of the potential utility of ACD-CPR in conjunction with an impedance threshold device rather than as an isolated therapy.

### 5.3. Mechanical Compression Devices

Mechanical devices have been developed that perform chest compressions and generate artificial circulation. There are two primary designs—one utilizes a load-distributing band applied circumferentially around the thorax, whereas the other uses a piston that is positioned over the sternum. Regardless of type, these devices have not been shown to improve outcomes from cardiac arrest and may be associated with an increased incidence of complications, most notably pneumothorax and hematoma [106,107,108,109,110,111]. While the data do not demonstrate superiority as compared to routine chest compressions during CPR, they (along with ACD-CPR devices) may play a vital role in sustaining high-quality chest compressions in the setting of too few rescuers, prolonged CPR, percutaneous intervention, or CPR required during transport [112,113]. Indeed, the use of a mechanical compression device was a major component of sustained CPR in all three major randomized trials evaluating eCPR [114,115,116].

### 5.4. Inspiratory Impedance Threshold Devices

An inspiratory impedance threshold device (ITD) is a valve connected to a patient undergoing bag-valve mask or invasive ventilation during chest compressions. When connected, air is precluded from entering the thorax during chest wall relaxation, thereby generating negative intrathoracic pressure, increasing venous return, augmenting preload, and improving myocardial perfusion [117]. Despite these favorable physiologic effects, ITD has not been shown to consistently improve clinical outcomes. In a large multicenter prospective trial of 8718 OHCA patients receiving traditional CPR with ITD or a sham device, there was no difference in survival to hospital discharge with satisfactory function (modified Rankin score ≤ 3) [118]. Similarly, there were no differences in other secondary outcomes, including the rates of ROSC, survival to hospital admission, or survival to hospital discharge. Studies investigating OHCA that employed both ACD-CPR and ITD therapy have shown that there may be a benefit in survival at 24 h, hospital discharge, and at 1-year follow-up [103,104,105,119]. Given these findings, the AHA 2020 CPR guidelines suggest that ITD may be considered in conjunction with ACD-CPR if equipment and trained personnel are available.

### 5.5. Interposed Abdominal Compression CPR

The addition of interposed abdominal compression (IAC) to standard CPR involves the application of positive pressure to the abdomen in opposition to the rhythm of chest compression. IAC-CPR serves to augment systolic and diastolic blood pressure, improving cerebral and myocardial perfusion [120]. When employed in an IHCA patient population, there is an increased rate of ROSC [121,122,123,124]. When evaluating its use by paramedics in an OHCA population, there was no significant difference in rates of resuscitation [125]. It remains to be seen whether IAC-CPR achieves improved rates of survival with a favorable functional status.

### 5.6. Automated Head/Thorax-Up Positioning CPR

The novel approach of automated head/thorax-up positioning CPR (AHUP-CPR) entails head and chest elevation to enhance venous return to augment cardiac preload, as well as allow for venous runoff from the intracranial space to avoid dangerous intracranial pressure elevation. Logistically, this is accomplished by the use of combined ACD-ITD, followed by the rapid deployment of an automated patient positioning device (APPD). The latter functions by elevating the patient’s head and mid-thorax to 12 cm and 8 cm, respectively. After 2 min, the patient’s head and thorax are raised to a final height of 24 cm and 12 cm.

AHUP-CPR has been primarily utilized in the OHCA patient population. In prospective registry studies, rapid initiation of AHUP-CPR conveyed a high probability of ROSC, survival to hospital discharge, and survival with favorable neurologic function in OHCA, irrespective of the presenting rhythm [126,127]. Follow-up prospective observational studies of AHUP-CPR in non-shockable OHCA demonstrated similar findings, with an increased likelihood of survival to hospital discharge in the AHUP-CPR group as opposed to the conventional CPR group [126,128].

### 5.7. Extracorporeal Cardiopulmonary Resuscitation (eCPR)

eCPR constitutes the utilization of VA-ECMO in order to restore cardiac output and systemic tissue perfusion. Its first successful application was by Kennedy in 1966, wherein seven out of eight patients (who were otherwise refractory to conventional resuscitative measures) were revived for a period of hours to days [129]. Given increasing provider familiarity with VA-ECMO (particularly for the management of cardiogenic shock) and greater availability of the technology, the use of eCPR has grown exponentially, surpassing 2000 cases annually [130].

To date, three major open-label, randomized controlled trials have assessed the efficacy of eCPR as opposed to standard ACLS. The ARREST trial was the first, randomizing 29 patients with refractory OHCA (requiring > 3 shocks) and initial shockable rhythms to eCPR versus standard ACLS [116]. At its first preplanned analysis, eCPR demonstrated superior survival to hospital discharge (43% vs. 7%, *p* = 0.023), and the trial was stopped early by the Data Safety and Monitoring Board (DSMB). Notably, favorable neurologic outcomes with a Cerebral Performance Category (CPC) 1–2 were higher in the eCPR group at discharge, 1 month, and 6 months; however, statistical comparisons could not be made due to a lack of survivorship in the ACLS arm. The Prague OHCA study enrolled 256 patients with a witnessed OHCA and any presenting rhythm without ROSC after 5 min and randomized them to intent to cannulate for eCPR or standard ACLS [114]. While this study was also stopped early by the DSMB, it was halted for futility. Survival at 180 days with a good neurologic outcome (CPC score 1–2) was not significantly different between groups (31.5% for eCPR versus 22.0% for ACLS; OR 1.63, 95% CI [0.93–2.85]; *p* = 0.09). Finally, the INCEPTION trial was the first multicenter eCPR study, enrolling 134 patients with an OHCA, initially shockable rhythm and absence of ROSC after 15 min to eCPR or standard ACLS [115]. Like the Prague OHCA trial, there was no difference in survival with a good neurologic status (CPC score 1–2) at 30 days between eCPR and ACLS (20% vs. 16%, respectively, *p* = 0.52). 

There is ongoing debate on how best to reconcile the results of the aforementioned. Some attribute the disappointing results from the Prague OHCA and INCEPTION trials to differences in trial design [131]. These include but are not limited to the patients selected (e.g., the type of rhythm included, duration of “low-flow” time until randomization), cannulation expertise and volume (e.g., longer times to cannulation in INCEPTION with lower rates of successful initiation), local infrastructure design (e.g., ARREST and Prague OHCA were single-center studies with long-standing and robust eCPR programs, whereas INCEPTION was across the entire Netherlands without specific eCPR protocols in every region), and the duration of neuroprognostication (e.g., median of 1 day in INCEPTION). Moreover, utilizing Bayesian analyses, both the Prague OHCA and INCEPTION trials demonstrated neurologically favorable survival at 180 days and 30 days, respectively, across a number of skeptical and enthusiastic scenarios [132,133]. Undoubtedly, more trials will be warranted to validate the utility of eCPR. However, initiation within any program will require considerable infrastructural development with emergency medical services, the cannulating “center”, and other local hospitals. Moreover, which patients are the most likely to benefit still has yet to be delineated.

**Table 1:** Select trials examining the outcomes of novel cardiopulmonary resuscitation strategies.

CPR Augmentation Strategy	Trial Design/Population	Primary Outcome	Results (Intervention versus Standard CPR)	Reference
*Active Compression–Decompression CPR*	Prospective, randomized control (*n* = 62)	Initial resuscitation	*62%* vs. *30%*, *p < 0.03*	Cohen TJ et al. [95]
	Single center	24 h survival	*45%* vs. *9%*, *p < 0.004*	
	IHCA	Hospital discharge GCS	*7%* vs. *0%*, *p = NS*	
			*8.0 ± 1.3* vs. *3.5 ± 0.3*, *p < 0.02*	
	Prospective, randomized control (*n* = 860)	ROSC	No significant differences in any outcome	Schwab TM et al. [101]
	Multicenter	Hospital admission		
	OHCA	Survival to discharge		
		Neurologic function		
	Prospective, randomized control	1 h survival (IHCA)	*35.1%* vs. *34.6%*, *p = 0.89*	Stiell IG et al. [102]
	Multicenter	Survival to discharge (IHCA)	*11.4%* vs. *10.4%*, *p = 0.64*	
	IHCA (*n* = 773)/OHCA (*n* = 1011)	MMSE (IHCA)	*37* vs. *37*	
		1 h survival (OHCA)	*16.5%* vs. *18.2%*, *p = 0.48*	
		Survival to discharge (OHCA)	*3.7%* vs. *4.6%*, *p = 0.49*	
		MMSE (OHCA)	*35* vs. *35*	
	Prospective, randomized control (*n* = 750)	1-year survival	*5%* vs. *2%*, *p = 0.03*	Plaisance P et al. [100]
	Multicenter			
	OHCA			
*Mechanical Compression Devices*	Prospective, randomized control (*n* = 2589)	4 h survival	*23.6%* vs. *23.7%*, *p > 0.99*	Rubertsson S et al., LINC trial [108]
	Multicenter			
	OHCA			
	Piston device			
	Prospective, randomized control (*n* = 4231)	Survival to hospital discharge	*9.4%* vs. *11.0%*, *OR 1.06 (95% CI 0.83–1.37)*	Wik L et al., CIRC trial [109]
	Multicenter			
	OHCA			
	Band device			
	Pragmatic, cluster randomized control (*n* = 127)	Proportion of eligible participants successfully randomized	*6%* vs. *7%*, *OR 0.86 (95% CI 0.64–1.15)*	Perkins GD et al., PARAMEDIC trial [107]
	Multicenter			
	IHCA			
	Piston device			
*Impedance Threshold Devices*	Prospective, randomized control (*n* = 400)	24 h survival	*22%* vs. *33%*, *p = 0.02*	Plaisance P et al. [117]
	Multicenter			
	OHCA			
	Prospective, randomized control (*n* = 8718)	Survival to hospital discharge with modified Rankin score ≤ 3	*5.8%* vs. *6.0%*, *p = 0.71*	Aufderheide TP et al. [118]
	Multicenter			
	OHCA			
*Interposed Abdominal Compression CPR*	Prospective, randomized control (*n* = 143)	ROSC	*49%* vs. *28%*, *p = 0.01*	Sack JB et al. [124]
	Single center	24 h survival	*33%* vs. *13%*, *p = 0.009*	
	IHCA			
	Prospective, randomized control (*n* = 135)	ROSC	*51%* vs. *27%*, *p = 0.007*	Sack JB et al. [123]
	Single center	24 h survival	*33%* vs. *13%*, *p = 0.02*	
	IHCA	Survival to hospital discharge	*25%* vs. *7%*, *p = 0.02*	
	Prospective, randomized control (*n* = 291)	ROSC	*31%* vs. *28%*, *p = NS*	Mateer JR et al. [125]
	Single center			
	OHCA			
*Automated Head/Thorax Up Positioning CPR*	Prospective, observational (*n* = 2322)	ROSC	*34.2%* vs. *17.9%*, *p < 0.0001*	Pepe PE et al. [127]
	Single center			
	OHCA			
	Prospective, observational (*n* = 227)	Survival to hospital discharge (initiated in <11 min)	*OR 3.28 (95% CI 1.55–6.92)*	Moore JC et al. [126]
	Multicenter	Survival to hospital discharge (initiated in <18 min)	*OR 1.88 (95% CI 1.03*, *3.44)*	
	OHCA			
	Prospective, observational (*n* = 706)	Survival to hospital discharge	*7.6%* vs. *2.8%*, *OR 2.84 (95% CI 1.35*, *5.96)*	Bachista KM et al. [128]
	Multicenter			
	OHCA			
*Extracorporeal CPR (eCPR)*	Prospective, randomized control (*n* = 29)	Survival to hospital discharge	*43%* vs. *7%*, *p = 0.023*	Yannopoulos D et al., ARREST trial [116]
	Single Center			
	OHCA			
	Prospective, randomized control (*n* = 256)	Survival with good neurologic outcome (CPC 1 or 2) at 180 days	*31.5%* vs. *22.0%*, *p = 0.09*	Belohlavek J et al., Prague OHCA trial [114]
	Single Center			
	OHCA			
	Prospective, randomized control (*n* = 134)	Survival with good neurologic outcome (CPC 1 or 2) at 30 days	*20%* vs. *16%*, *p = 0.52*	Suverein MN et al., INCEPTION trial [115]
	Multicenter			
	OHCA			

Values are means ± SD unless otherwise reported. Abbreviations: CI, confidence interval; CPC, cerebral performance category; CPR, cardiopulmonary resuscitation; IHCA, in-hospital cardiac arrest; OHCA, out-of-hospital cardiac arrest; OR, odds ratio; ROSC, return of spontaneous circulation.

## 6. Conclusions

Despite the tremendous progress made in the development of CPR as we know it today, there is still much to learn on how best to monitor and augment systemic perfusion during cardiac arrest. While novel techniques such as the ones described continue to be innovated and studied, it is imperative that we continue to teach and reinforce the basics—high-quality chest compressions while maintaining adequate ventilation and minimal interruptions. This still remains our most readily and widely available asset in the treatment of cardiac arrest. 

## Data Availability

No new data were created or analyzed in this study.

## Abbreviations

ACLS	Advanced cardiac life support.
ACD-CPR	Active compression–decompression cardiopulmonary resuscitation
ACD-ITD	Active compression–decompression—impedance threshold device.
AHUP-CPR	Automated head-up position—cardiopulmonary resuscitation.
AMC	Area of maximal compression.
CPR	Cardiopulmonary resuscitation.
CO_2_	Carbon dioxide.
DBP	Diastolic blood pressure.
eCPR	Extracorporeal cardiopulmonary resuscitation.
ETCO_2_	End-tidal carbon dioxide.
IAC	Interposed abdominal compressions.
ITD	Impedance threshold device.
LV	Left ventricle.
LVOT	Left ventricular outflow tract.
NIRS	Near-infrared spectroscopy.
OHCA	Out-of-hospital cardiac arrest.
ROSC	Return of spontaneous circulation.
rSO2	Regional cerebral oximetry.
TEE	Transesophageal echocardiogram.
TTE	Transthoracic echocardiogram.
VA-ECMO	Venoarterial extracorporeal membrane oxygenation.
VF	Ventricular fibrillation.
